# ****β****-Cell Specific Overexpression of GPR39 Protects against Streptozotocin-Induced Hyperglycemia

**DOI:** 10.1155/2011/401258

**Published:** 2011-11-17

**Authors:** Kristoffer L. Egerod, Chunyu Jin, Pia Steen Petersen, Nils Wierup, Frank Sundler, Birgitte Holst, Thue W. Schwartz

**Affiliations:** ^1^Laboratory for Molecular Pharmacology, Department of Neuroscience and Pharmacology, University of Copenhagen, Blegdamsvej 3, 2200 Copenhagen, Denmark; ^2^Section for Metabolic Receptology and Enteroendocrinology, Novo Nordisk Foundation Center for Basic Metabolic Research, University of Copenhagen, Blegdamsvej 3, 2200 Copenhagen, Denmark; ^3^Division of Diabetes, Metabolism, and Endocrinology, Department of Experimental Medical Science, Lund University, Lund, Sweden

## Abstract

Mice deficient in the zinc-sensor GPR39, which has been demonstrated to protect cells against endoplasmatic stress and cell death *in vitro*, display moderate glucose intolerance and impaired glucose-induced insulin secretion. Here, we use the Tet-On system under the control of the proinsulin promoter to selectively overexpress *GPR39* in the **β** cells in a double transgenic mouse strain and challenge them with multiple low doses of streptozotocin, which in the wild-type littermates leads to a gradual increase in nonfasting glucose levels and glucose intolerance observed during both food intake and OGTT. Although the overexpression of the constitutively active GPR39 receptor in animals not treated with streptozotocin appeared by itself to impair the glucose tolerance slightly and to decrease the **β**-cell mass, it nevertheless totally protected against the gradual hyperglycemia in the steptozotocin-treated animals. It is concluded that GPR39 functions in a **β**-cell protective manner and it is suggested that it is involved in some of the beneficial, **β**-cell protective effects observed for Zn^++^ and that GPR39 may be a target for antidiabetic drug intervention.

## 1. Introduction

GPR39 is a member of the ghrelin receptor family, all of which are peptide receptors described to be involved in the peripheral and/or central control of appetite, GI tract function, energy homeostasis, and metabolism [1–5]. The *GPR39* locus is rather complex with an overlapping antisense gene LYPD1 and the occurrence of alternative splicing [6]. Importantly, however, the full-length, functional GPR39 receptor is expressed exclusively in peripheral, endocrine, and metabolic organs such as the endocrine pancreas, the liver, the kidney, the GI tract, and the white adipose tissue [6, 7].

GPR39 functions as a zinc sensor being activated by physiological concentrations of Zn^++^ which is particularly interesting in the pancreatic islets where Zn^++^ is released in relatively large amounts together with insulin [8, 9]. Although it was reported that a peptide fragment of the ghrelin precursor called obestatin could act as an agonist for GPR39 [10], this could not be confirmed [11–14] and the original report was later retracted [15]. As observed for key members of this receptor family such as the ghrelin receptor and the neurotensin NT2 receptor, GPR39 signals with high ligand independent or constitutive activity [2, 3]. This is observed in the Gq pathway as measured by inositol phosphate accumulation and, for example, in activation of serum-responsive-element- (SRE-) regulated transcriptional activity mainly mediated through the G12/13 pathway [2].

Unchallenged *Gpr39*-deficient mice have a rather modest overall phenotype [7, 14]. However, more careful studies both by Tremblay and coworkers and by our group revealed that GPR39 deficiency is associated with *β*-cell dysfunction including decreased expression of key regulatory genes and impaired glucose-induced insulin secretion, for example from isolated perifused pancreatic islets as well as moderate glucose intolerance *in vivo* [16, 17]. The mechanism by which GPR39 is important for *β*-cell function is unclear; however, it could be related to the recently described, general cell protective effect of GPR39 [18].

In the present study, we exploit the Tet-On system to create a “knock-in” transgenic mouse strain with inducible overexpression of human *GPR39* selectively in pancreatic *β* cells. The mice express the human *GPR39* gene under the control of the tetracycline operator and the reverse tetracycline-controlled transactivator (rtTA) driven by the rat insulin promoter (RIP) [19, 20]. Because GPR39 displays a high degree of constitutive activity an increased expression of GPR39 will be directly associated with an increased receptor signaling activity in the *β* cells independently of an endogenous ligand.

## 2. Materials and Methods

### 2.1. The Transgenic Mouse

**Figure 1:**
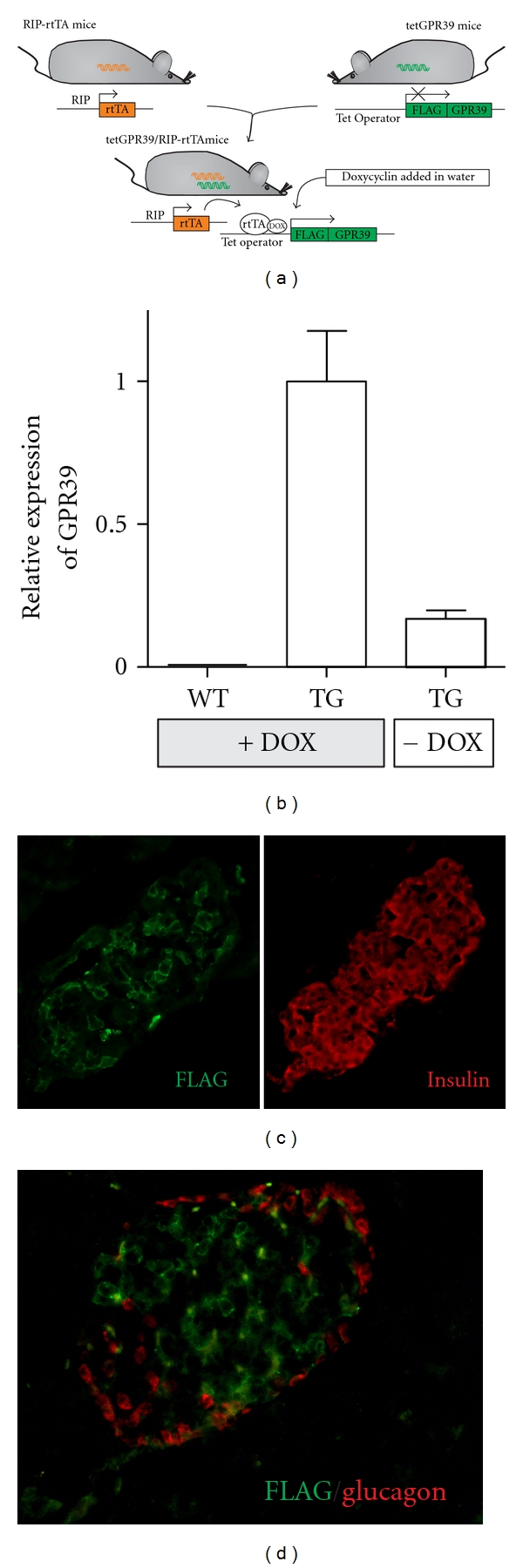
Overview of the generation of the tetGPR39/RIP-rtTA transgenic mice and demonstration of the *β*-cell specific expression of GPR39. (a) Schematic diagram of the generation of the tetGPR39/RIP-rtTA transgenic mice by crossing of the RIP-rtTA mouse with the tetGPR39 mouse. (b) QPCR specific for the transgenic FLAG-tagged human GPR39 performed on whole pancreas. (c, d) Immunohistochemistry of pancreatic islets from tetGPR39/RIP-rtTA transgenic mice, in (c, d) GPR39 was detected using the M1 anti-FLAG antibody (green). (c) Antiinsulin antibody (red). (d) Antiglucagon antibody (red).

B6-TgH(tetGPR39/RIP-rtTA) transgenic mice were generated by crossing heterozygous RIP-rtTA transgenic mice (kindly provided by Dr. Yuval Dor) with heterozygous tetGPR39 transgenic mice (Figure 1). The tetGPR39 mice were acquired from Nucleis, and briefly a construct consisting of an N-terminal FLAG tag linked to the total coding region for human *GPR39* followed by the SV40 polyA signal was inserted into the HPRT locus through target homologous recombination.

The double transgenic mice were backcrossed into C57BL/6 for at least three generations and littermates were used as control.

To verify doxycycline- (DOX-) induced GPR39 expression, 3 tetGPR39/RIP-rtTA transgenic mice and 6 WT littermates were given DOX in the drinking water (1 mg/mL), whilst 3 tetGPR39/RIP-rtTA mice were given normal water (mock) to evaluate leakiness of the system. After 6 days of DOX or mock treatment, mice were sacrificed and pancreas was isolated for immunohistochemistry and real-time quantitative PCR (QPCR).

### 2.2. Outline for the Experimental Setup

Mice were treated with multiple low-dose STZ to induce hyperglycemia [21]. Briefly, intraperitoneal injection (IP) of 40 mg/kg STZ (diluted in citric acid pH = 4) was carried out on five consecutive days.

Two groups consisting of 11 tetGPR39/RIP-rtTA transgenic mice and 11 wild-type littermates were treated with low-dose STZ injections. In parallel two groups consisting of 7 tetGPR39/RIP-rtTA transgenic mice and 6 wild-type littermates were mock treated with vehicle injections for 5 days. DOX treatment was initiated 3 days prior to the injection and maintained throughout the whole experiment for all four groups.

Using a glucometer (Ascensia Elite XL Diabetes Care System, Bayer HealthCare) nonfasting blood glucose was measured twice a week in tail blood to follow the development of hyperglycemia. The nonfasting blood glucose levels were analyzed by repeated measures (mixed model) ANOVA using GraphPad Prism version 5.0a. 

At day 30, blood glucose and insulin levels in response to feeding were measured. Mice were fasted for 16 hours prior to *ad libitum* feeding whilst tail blood was obtained at times −30, 0, 20, 40, and 85 minutes to monitor blood glucose levels, and blood from the orbital sinus was collected at times 0, 20, and 40 minutes to measure plasma insulin levels using the Sensitive Rat Insulin RIA kit (Millipore). Insulin and glucose levels were analyzed by repeated measures (mixed model) ANOVA using GraphPad Prism version 5.0a.

At day 35, oral glucose tolerance test (OGTT) was performed, glucose (1.5 g/kg body weight) was administered by oral gavage, at times −30, 0, 10, 20, 30, 60, 90, and 120, minutes and blood glucose levels were measured in tail blood using a glucometer. Glucose levels were analyzed by repeated measures (mixed model) ANOVA and by a Mann-Whitney *t*-test using GraphPad Prism version 5.0a.

At day 40, the mice were sacrificed and pancreas was exsected for immunocytochemistry.

All mice used in this study were male; they were housed in a light/dark cycle of 12 hours with free access to food and water. All animal studies were conducted in accordance with international guidelines and approved by the Animal Experiments Inspectorate in Denmark, which follows the EU (86/609/EEC) guidelines.

### 2.3. QPCR

QPCR was performed using the Mx3000P (Stratagene), and the SYBR Premix Ex Taq (Takara) was used for SYBR green-based QPCR. Cycle threshold values were obtained using Stratagene Mx3000P software, and the delta-delta Ct method was used to calculate the relative fold change of RNA levels compared to a calibrator sample. Tyrosine 3-monooxygenase/tryptophan 5-monooxygenase activation protein, zeta polypeptide (Ywhaz) was used as reference gene. Primer sequences for human GPR39, mice Gpr39, and Ywhaz were 5′-TCC TGA GGC TGA TTG TTG TG-3′, 5′-GTG TAC AGG AGC GGG TTG AT-3′, 5′-AGT GAG GAG AGC CGG ACA G-3′, 5′-CAG TCA TGT TTG GGT TTT GC-3′, 5′-AGA CGG AAG GTG CTG AGA AA-3′ and 5′-GAA GCA TTG GGG ATC AAG AA-3′, respectively.

Exsected tissue was snap-frozen in liquid nitrogen and stored at −80°C until RNA was extracted using the SV Total RNA Isolation System (Promega) followed by cDNA synthesis using the ImProm-II Reverse Transcriptase (Promega). 

### 2.4. Immunohistochemistry and Morphometry

The protocol has been reported [22]. Briefly, pancreata were fixed in Stefanini's solution (2% formaldehyde and 0.2% picric acid in 0.1 M PBS (pH 7.2)), rinsed in sucrose-enriched (10%) buffer, and frozen on dry ice. Thereafter sections were cut (10 *μ*m) and mounted on slides. The sections were stained for insulin (code 9003, dilution 1 : 1280; Euro-Diagnostica, Malmö, Sweden), glucagon (code 7811, dilution 1 : 5120; Euro-Diagnostica), and FLAG (code F7425, dilution 1 : 800, Sigma-Aldrich) as previously [16].

To quantify *β*-cell area, 4 sections were taken through the whole pancreas, with a distance of >100 *μ*m between each, according to the previously published protocols [23]. Thereafter images of all immunostained islets in each section were taken with a digital camera (Nikon DS-2Mv). The stained area was measured using BioPix iQ 2.0 software (BioPix AB, Göteborg, Sweden). *β*-cell areas were analyzed by a Mann-Whitney *t*-test using GraphPad Prism version 5.0a.

## 3. Results

The double transgenic mice strain B6-TgH(tetGPR39/RIP-rtTA) displayed tetracycline, that is, DOX inducible expression of the FLAG-tagged human *GPR39* selectively in the pancreatic *β* cells as determined by immunohistochemistry after 6 days of DOX treatment (Figure 1). As often described in general and specifically for the proinsulin-promoter-driven Tet-On system some degree of leakiness was observed [24, 25]. In our case the leakiness corresponded to one fifth of the expression of the human *GPR39* in the pancreas before DOX administration (Figure 1(b)). In accordance with this, wild-type littermates were used as controls in the functional studies.

**Figure 2:**
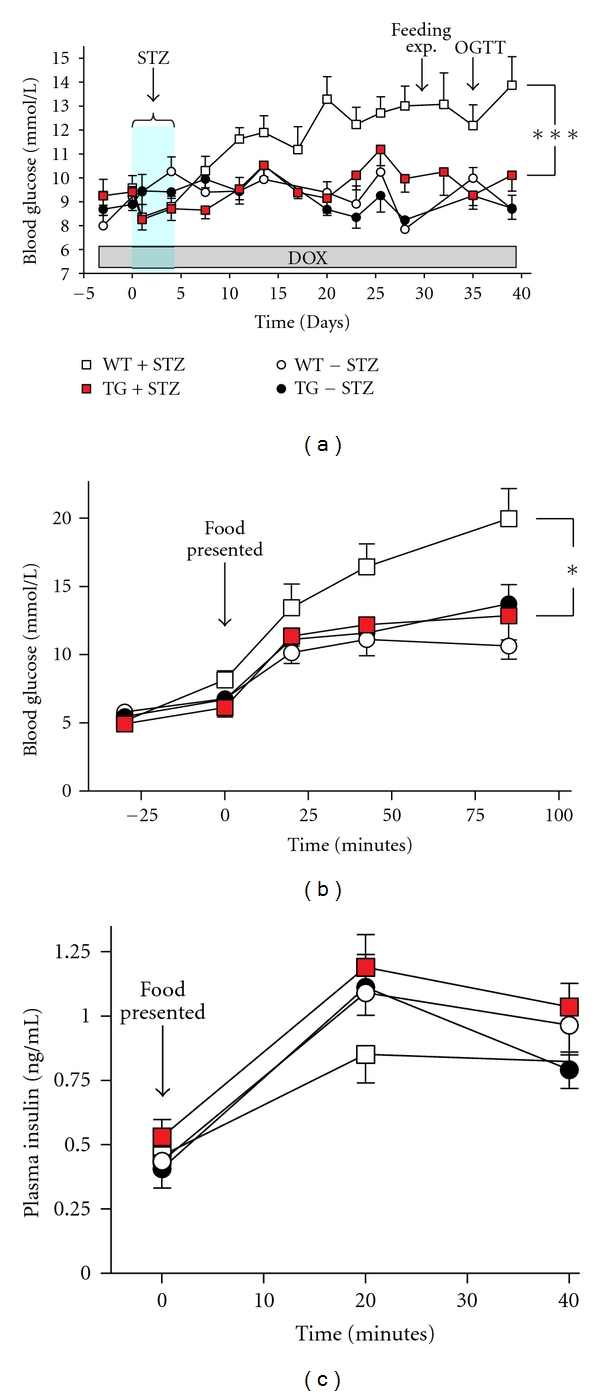
Protection against STZ-induced hyperglycemia by *β*-cell specific overexpression of GPR39 in the tetGPR39/RIP-rtTA mice. (a) Nonfasting blood glucose levels measured twice a week. DOX was given in the drinking water to all four groups of animals from day 3. A group of tetGPR39/RIP-rtTA transgenic mice (TG + STZ (red square), *n* = 11) and a group of wild-type littermates (WT + STZ (open square), *n* = 11) were treated with low-dose STZ injections, 40 mg/kg daily from day 0 to day 4. A group of tetGPR39/RIP-rtTA transgenic mice (TG − STZ (black circles), *n* = 7) and a group of wild-type littermates (WT − STZ (open circles), *n* = 6), were mock treated with vehicle injections from day 0 to day 4. (b, c) At day 28, all animals were fasted for 18 hours before given access to food at time 0: the blood glucose (b) and insulin (c) were monitored at times indicated. *P < 0.05, ***P < 0.001 (repeated measures (mixed model) ANOVA performed on WT + STZ versus TG + STZ).

Repeated low doses of STZ normally induce a gradual damage of *β* cells resulting in nonfasting hyperglycemia [21, 26, 27]. As shown in Figure 2(a), treatment of wild-type animals with 40 mg/kg STZ for five days as expected resulted in an increase in nonfasting glucose appearing after approximately 10 days (open squares) as compared to vehicle-treated animals (open circles)—all receiving DOX in the drinking water throughout the experiment. In contrast, no clear increase in blood glucose was observed after STZ treatment in the *GPR39* transgenic animals (Figure 2(a), red squares). When compared to the STZ treated wild-type littermates (open squares) the GPR39 transgenic mice displayed lower blood glucose throughout the experiment (*P* = 0.0007, *n* = 11, two-way ANOVA).

A controlled feeding experiment was performed on day 30, which demonstrated that fasting blood glucose was normal and similar in all four groups, that is, around 5 mmol/L (Figure 2(b)). However, postprandial hyperglycemia was observed in the STZ treated wild-type animals as compared to the STZ-treated *GPR39* transgenic littermates after presentation of food and eating *ad libitum* (*P* = 0.037, *n* = 11, two way ANOVA). In fact, the STZ-treated *GPR39* transgenic mice displayed a postprandial glucose increase similar to that observed in the two non-STZ-treated control groups (Figure 2(b)). The fasting insulin levels were also similar in all four groups of animals (Figure 2(c)). Although the postprandial serum insulin 20 min. after food intake apparently was lower in the STZ-treated wild-type animals (0.86 ± 0.11 ng/mL, *n* = 11) compared to the non-STZ treated control group (1.10 ± 0.09 ng/mL, *n* = 6), this did not reach statistical significance (*P* = 0.15, *t*-test). Importantly, just like the glucose level the serum insulin level of the STZ-treated transgenic *GPR39* animals was similar to that of the non-STZ-treated control groups (1.2 ± 0.13 ng/mL, *n* = 11, Figure 2(c), red squares).

**Figure 3:**
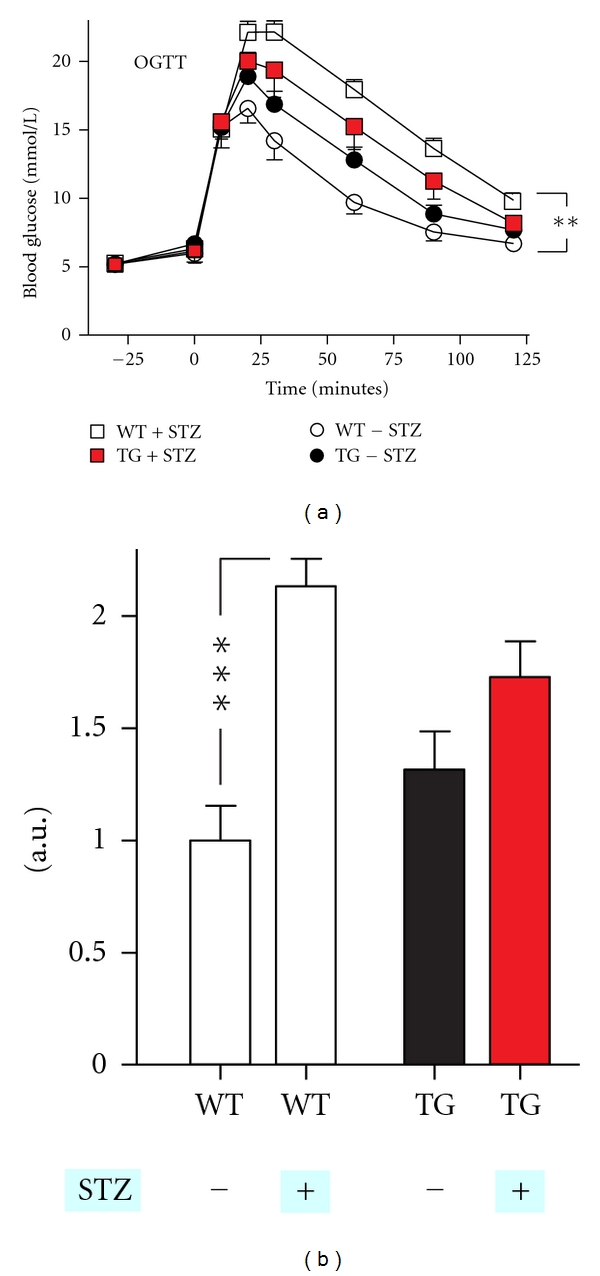
Effect of *β*-cell specific overexpression of GPR39 on oral glucose tolerance test (OGTT) after low-dose STZ treatment. (a) OGTT performed 35 days after STZ treatment (see Figure 2) in tetGPR39/RIP-rtTA transgenic (TG) mice with STZ treatment (red square) *n* = 11, in wild-type littermates (WT) with STZ treatment (open squares) *n* = 11, in WT mock-treated mice (open circles) *n* = 6, and in TG mock-treated mice (black circles) *n* = 7. **P < 0.01 (repeated measures (mixed model) ANOVA performed WT + STZ versus WT − STZ). (b) area under the curve, ***P < 0.001 (Mann-Whitney *t*-test). a.u.: arbitrary units.

OGTT performed on day 35 revealed that the low-dose STZ treatment as expected rendered the wild-type mice glucose intolerant but not diabetic as their fasting glucose levels were normal (Figure 3(a), open squares versus open circles)—as also observed during the food test (Figure 2(b)). The transgenic over expression of *GPR39* in the *β* cells by itself seemed to impair the glucose tolerance as determined during the OGTT (Figure 3(a), black versus open circles). Nevertheless, the glucose excursions of the STZ-treated *GPR39* transgenic animals appeared to be lower as compared to their STZ-treated wild-type littermates (Figure 3(a), red squares versus open squares). Except for the effect of STZ on the wild-type animals none of these differences reached statistical significance.

**Figure 4:**
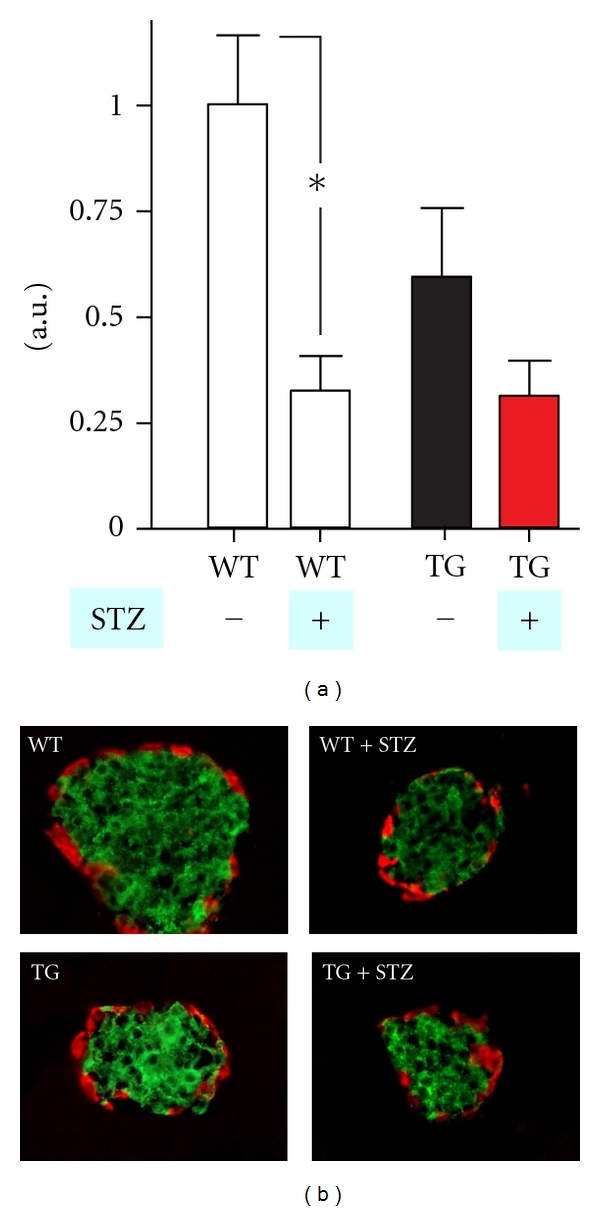
Quantification of the *β*-cell area in pancreatic islets by immunohistochemistry. (a) WT mock-treated mice (empty bars − STZ) *n* = 7 and tetGPR39/RIP-rtTA transgenic (TG) mock-treated mice (black filled bars − STZ) *n* = 6. WT STZ treated (empty bars + STZ) *n* = 5, tetGPR39/RIP-rtTA transgenic (TG) STZ-treated mice (red filled bars + STZ) *n* = 5. *P < 0.05 (Mann-Whitney *t*-test). (b) Representative islets stained with anti-insulin (green) and anti-glucagon antibodies (red). a.u.: arbitrary units.

Immunohistochemical analysis after 40 days of treatment demonstrated a similar but opposite pattern of effects of STZ treatment on *β*-cell area as observed for blood glucose during the OGTT. That is, STZ severely reduced the *β*-cell area to 32% in the wild-type animals (*P* = 0.005, *n* = 11 STZ treated, *n* = 6 mock treated, Mann Whitney), and, like the transgenic overexpression of *GPR39* in itself impaired the glucose tolerance, it apparently also reduced the *β*-cell area, that is, to approximately 60%—although the effect was not statistically significant (Figure 4). The area of *β*-cells determined in the pancreas after STZ treatment of the transgenic animals was similar to that observed in the STZ-treated wild-type littermates. Thus, the relative effect of STZ was smaller in the GPR39 transgenic animals than in the wild-type animals.

## 4. Discussion

It has previously been demonstrated that *Gpr39* deficiency leads to impaired *β*-cell function with decreased glucose induced insulin secretion and moderate glucose-intolerance [16, 17].*GPR39* was in the present study overexpressed in a *β*-cell specific, inducible manner in a double transgenic strain of mice using the Tet-On system under the control of the proinsulin promoter. Although the overexpression in itself appeared to affect the *β*-cell mass and impaired the glucose tolerance slightly, it nevertheless totally protected against the gradual hyperglycemia observed after low-dose STZ treatment of wild-type littermates. Thus, the zinc-sensor GPR39 appears to function in a *β*-cell protective manner and could accordingly be a target for antidiabetic drug intervention. It was recently shown by transgenic expression of a specifically designed 7TM receptor, which could be activated by a pharmaceutical compound that acute, selective G*α*_q_ activation in the *β* cells leads to improved glucose tolerance and increased insulin secretion [28]. Since the GPR39 receptor signals mainly through G*α*_q_ [2] and is normally expressed in the *β* cells, the observation of Guettier and coworkers together with the previous studies in *Gpr39*-deficient mice [16, 17] and the present study with *β*-cell specific overexpression of *GPR39* all support the notion that GPR39 could function as an interesting antidiabetic drug target.

**GPR39 and Zinc in Islet Protection**

GPR39 was originally discovered by simple homology screening for novel members of the ghrelin receptor family [29]. Interestingly, however, GPR39 was recently “rediscovered” as one out of two, highly upregulated 7TM receptors in a naturally occurring, apoptosis-resistant cell line [18]. Subsequent transfection of GPR39 into other cell lines protected these cells against oxidative and endoplasmatic reticulum stress, which was not the case with the other receptors [18]. The notion that the zinc-sensor GPR39 particularly in the endocrine pancreas could be involved in the adaptive, protective response to stress and cell damage is supported by the fact that apoptosis of pancreatic islet cells is associated with the release of intracellular storages of zinc from the dying cells [30]. Importantly, as we in the present study find that *GPR39* overexpression protects against the development of nonfasting hyperglycemia, zinc-sulfate-enriched drinking water has been reported to prevent STZ-induced diabetes in mice [31, 32] and, importantly, to protect against the spontaneous development of diabetes in both NOD mice and biobreeding rats [31, 33]. It is tempting to suggest that the zinc-sensor GPR39 with its cell protective properties could be involved in some of these beneficial effects of zinc. However, GPR39 may not only protect islets from apoptosis but may also be involved in proliferative, adaptive responses of the islets as Tremblay and coworkers found that *Gpr39*-deficient mice do not display the normal pancreatic islet hyperplasia in response to diet-induced obesity [17]. In view of the documented islet protective properties of *GPR39*, an agonist of GPR39 could potentially be useful in the treatment of diabetes where the *β* cells are under oxidative stress [34, 35].

**Transgenic Overexpression as a Model of Agonist Action**

Overexpression of *GPR39* is in the present study employed as a tool trying to mimic a situation with continuous GPR39 agonist treatment. An issue in this approach in general is whether you obtain appropriate receptor signaling and in the appropriate situation. With *GPR39* we do obtain a protection of the *β* cells when they are challenged as judged from the prevention of the increase in nonfasting blood glucose after STZ treatment (Figure 2). However, the transgenic overexpression of *GPR39* apparently in itself impairs glucose tolerance and affects *β*-cell mass—albeit not significantly (Figures 3 and 4). This situation, where a signal obtained through transgenic overexpression can function as both “friend and foe,” is not atypical and has, for example, been suggested for the G protein subunit *α*_q_(G*α*_q_) where the effect of the signaling also is balancing between promoting cell survival and apoptosis [36, 37].This could be relevant for the GPR39 receptor which signals through G*α*_q_ but probably also through G12/13 [2]. However, because GPR39 displays high constitutive signalling, the increased transgenic expression of the receptor will in itself directly lead to increased signaling activity independent of the presence of agonist. In other words, it is possible that a high level of receptor expression will lead to an inappropriate degree of signaling in the unchallenged situation, that is, when the *β* cells are not in a stress situation. Likewise, counter regulatory effects from constitutively Gs signaling in the *β* cells have been observed [28]. Post-transcriptional regulation renders the correlation of RNA to protein very complicated even when comparing the same RNA transcript under various conditions [38, 39]. Thus, predicting protein levels based on the two very different RNA transcripts endogenous *Gpr39* and the transgenic *GPR39* is inherently flawed. So although the expression level of the transgenic, human *GPR39* appears to be rather moderate, that is, only approximately 1.5-fold on top of the endogenous mouse *Gpr39* expression (data not shown), the protein level could be much higher. At least it appears that care should be taken in increasing GPR39 signaling in the *β* cells in the unchallenged situation as this may impair glucose tolerance in itself. However, at the end of the day when pharmacological agonist tools for GPR39 are available it may simply be a question of using the right dose and treating at the right time.

## Abbreviations

7TM: Seven transmembrane
rtTA: Reverse tetracycline-controlled transactivator
STZ: Streptozotocin
WT: Wild type
DOX: Doxycycline
YWHAZ: Tyrosine 3-monooxygenase/tryptophan 5-monooxygenase activation protein, zeta polypeptide
OGTT: Oral glucose tolerance test.
